# Vitrectomy combined with intravitreal antifungal therapy for posttraumatic fungal endophthalmitis in eastern China

**DOI:** 10.1186/s12886-020-01703-7

**Published:** 2020-11-03

**Authors:** Hong Zhuang, Xinyi Ding, Ting Zhang, Qing Chang, Gezhi Xu

**Affiliations:** 1 Department of Ophthalmology, Eye & ENT Hospital, Fudan University, 83 Fenyang Road, Shanghai, 200031 China; 2 Shanghai Key Laboratory of Visual Impairment and Restoration, and NHC Key Laboratory of Myopia (Fudan University), Shanghai, 200031 China

**Keywords:** Fungal endophthalmitis, Penetrating ocular trauma, Vitrectomy, Antifungal therapy

## Abstract

**Background:**

To evaluate the effect and prognostic factors of vitrectomy combined with intravitreal antifungal therapy for posttraumatic fungal endophthalmitis in Eastern China.

**Methods:**

We retrospectively reviewed the medical records of patients who developed fungal endophthalmitis after penetrating ocular trauma at an ophthalmic center in Eastern China. All patients underwent vitrectomy and intravitreal injection of antifungal drugs.

**Results:**

Thirty-five patients (35 eyes) were included. Twelve eyes suffered plant trauma, 17 eyes metal trauma, and 6 eyes other trauma. The culture results for all 35 eyes showed filamentous fungi, including *Aspergillus* in 26 eyes (74.3%). Twenty-three eyes underwent vitrectomy once and 12 eyes were treated twice. Four eyes were iridectomized because of a fungal lesion behind the iris. Fungal endophthalmitis was effectively controlled in 33 eyes (94.3%), whereas 2 eyes were ultimately enucleated. Visual acuity was significantly better after treatment than before treatment (*P* = 0.0006). According to the preoperative vision, the affected eyes were divided into two groups: group 1A (light perception) and group 1B (better than light perception). The final visual acuity in group 1B was significantly better than that in group 1A (*P* = 0.0289).

**Conclusions:**

Vitrectomy combined with intravitreal antifungal therapy is an effective treatment for posttraumatic fungal endophthalmitis. Preoperative visual acuity is a significant factor affecting the prognosis of visual acuity.

**Supplementary Information:**

The online version contains supplementary material available at 10.1186/s12886-020-01703-7.

## Background

Fungal endophthalmitis is a devastating infectious disease that can lead to serious visual impairment or even the loss of an eye. It can be divided into endogenous and exogenous endophthalmitis, which have completely different routes of infection. Endogenous fungal endophthalmitis is caused by the hematogenous spread of infectious microbes from distant foci and usually has systemic risk factors [1–3]. Exogenous fungal endophthalmitis is caused by pathogens brought directly into the eye by an open eye injury or intraocular surgery [4], or is secondary to fungal keratitis [5].

The prevalence of posttraumatic fungal endophthalmitis varies in different countries. Fungal endophthalmitis after trauma predominantly occurs in developing countries, such as India and China [6–8], but it is rare in developed countries. A report from the USA retrospectively reviewed 41 cases of fungal endophthalmitis over 16 years, which included just 10 cases of posttraumatic fungal endophthalmitis [9]. Although there have been a few reports of fungal endophthalmitis after trauma, there are no established standard therapies. The treatment of fungal endophthalmitis is very challenging. In this study, we evaluate the effect of vitrectomy combined with intravitreal antifungal therapy for posttraumatic fungal endophthalmitis in Eastern China. Meanwhile, we analyzed the prognostic factors for postoperative visual outcomes.

## Methods

This research adhered to the tenets of the Declaration of Helsinki and was approved by the Ethics Committee of the Eye and ENT Hospital of Fudan University (Shanghai, China). Written informed consent was obtained from each patient after the nature and possible consequences of the study had been explained.

The Eye and ENT Hospital of Fudan University is a tertiary hospital that admits patients with endophthalmitis from provinces in eastern China. We retrospectively reviewed all patients who developed fungal endophthalmitis after penetrating ocular trauma who attended the hospital between May 2014 and December 2019. During the study period, there were 586 cases of posttraumatic endophthalmitis, of which 35 cases were confirmed as fungal endophthalmitis (accounting for 6.0%).

Fungal endophthalmitis was diagnosed according to the ocular manifestations of endophthalmitis after trauma and the results of microbial cultures of intraocular fluid (aqueous humor or vitreous). The medical records of the patients were reviewed to obtain their demographic data, onset features, pathogenic organisms, treatments, and best-corrected visual acuity. According to the report of the ocular trauma classification group [10], the zone of penetrating injuries was recorded. All patients underwent comprehensive ophthalmic examinations, including slit-lamp biomicroscopy, examination of ocular fundus, and B-scan ultrasonography.

In the process of treatment, all patients underwent vitrectomy and intravitreal injection of an antifungal drug (5 μg of amphotericin B or 100 μg of voriconazole). Pars plana vitrectomy with three incisions was performed to remove the inflamed vitreous. During surgery, samples of the aqueous humor and vitreous were collected for microbial culture. The decision to combine vitrectomy with lensectomy or iridectomy was based on the preoperative examination of the patient and with consideration of the intraoperative conditions.

We analyzed the difference of visual acuity before and after treatment. And we analyzed the factors that influenced the visual outcome. The statistical analysis was performed using Stata 11.0 statistical software (Stata Corporation, College Station, TX, USA). The Cochran–Mantel–Haenszel χ^2^ test was used to compare visual acuity between two groups. A *P* value of < 0.05 was considered statistically significant.

## Results

### Clinical features and culture results

The 35 patients (35 eyes) included 27 males and eight females, with ages ranging from 7 to 69 years and a mean age of 45.6 ± 17.2 years (median age 48 years). The patients were followed up for 3–19 months, and the mean follow-up period was 8.1 ± 3.9 months (median 8 months). Trauma was caused by a plant in 12 eyes, a metal object in 17 eyes (iron objects in 16 eyes), and other factors in six eyes. There were 27 eyes of penetrating trauma in Zone I and 8 eyes in Zone II. Ocular trauma caused lens damage in 20 eyes.

The time from trauma to diagnosis of endophthalmitis was < 1 week in two patients (5.7%), 1–2 weeks in five patients (14.3%), from 2 weeks to 1 month in 13 patients (37.1%), and > 1 month in 15 patients (42.9%). The culture results for all 35 eyes showed filamentous fungi (molds), including *Aspergillus* in 26 eyes (74.3%), other fungi in four eyes (11.4%) (*Fusarium*, *Paecilomyces*, *Mucor*, and Dematiaceous mold), and unidentified molds in five eyes (14.3%). Table 1 lists the demographic data, onset features, and culture results.

**Table 1:** Demographic data, onset features, and culture results of patients with fungal endophthalmitis

Patient No.	Sex	Age (years)	Penetrating object	Time from trauma to endophthalmitis	Pathogenic organism
1	M	39	Plant (chestnut thorn)	3 months	*Aspergillus*
2	M	57	Plant (chestnut thorn)	26 days	*Aspergillus*
3	F	64	Plant (chestnut thorn)	70 days	*Aspergillus*
4	M	22	Plant (bamboo stick)	3 months	*Paecilomyces lilacinus*
5	M	10	Plant (bamboo stick)	4 days	*Aspergillus*
6	F	46	Plant (bamboo stick)	20 days	*Aspergillus*
7	M	58	Plant (tree branch)	28 days	Unidentified mold
8	M	66	Plant (tree branch)	26 days	*Fusarium*
9	M	49	Plant (tree branch)	18 days	*Aspergillus*
10	F	60	Plant (wood stick)	2 months	*Aspergillus*
11	M	67	Plant (wood stick)	35 days	*Aspergillus*
12	M	57	Plant (wood stick)	9 days	Unidentified mold
13	M	44	Metal (iron scurf)	35 days	*Aspergillus*
14	M	7	Metal (iron scurf)	3 days	Unidentified mold
15	M	49	Metal (iron nail)	40 days	*Aspergillus*
16	M	27	Metal (iron nail)	40 days	Unidentified mold
17	M	20	Metal (iron wire)	18 days	*Aspergillus*
18	M	33	Metal (iron wire)	16 days	*Aspergillus*
19	M	48	Metal (iron wire)	25 days	*Aspergillus*
20	M	31	Metal (iron wire)	12 days	*Mucor*
21	M	64	Metal (iron wire)	38 days	Aspergillus
22	M	58	Metal (iron wire)	22 days	*Aspergillus*
23	F	8	Metal (iron wire)	28 days	Dematiaceous mold
24	M	69	Metal (iron wire)	12 days	*Aspergillus*
25	M	47	Metal (iron wire)	7 days	*Aspergillus*
26	F	47	Metal (iron wire)	20 days	*Aspergillus*
27	M	34	Metal (iron wire)	35 days	*Aspergillus*
28	M	57	Metal (iron wire)	11 days	*Aspergillus*
29	M	43	Metal (copper key)	2 months	*Aspergillus*
30	M	47	Other (plastic object)	40 days	*Aspergillus*
31	F	40	Other (plastic object)	75 days	*Aspergillus*
32	F	53	Other (plastic object)	4 months	Unidentified mold
33	M	52	Other (brick fragments)	26 days	*Aspergillus*
34	F	54	Other (ceramic fragments)	4 months	*Aspergillus*
35	M	69	Other (crushed stone)	25 days	*Aspergillus*

Preoperative examination of the anterior segment showed that all the affected eyes had inflammatory exudation in the anterior chamber, and B-scan ultrasonography showed obvious vitreous inflammation. Hypopyon was detected in 28 eyes (80.0%) and the hypopyon was always sticky. Four eyes (11.4%) had mass lesions in the anterior chamber.

### Treatments and visual prognosis

**Fig. 1:**
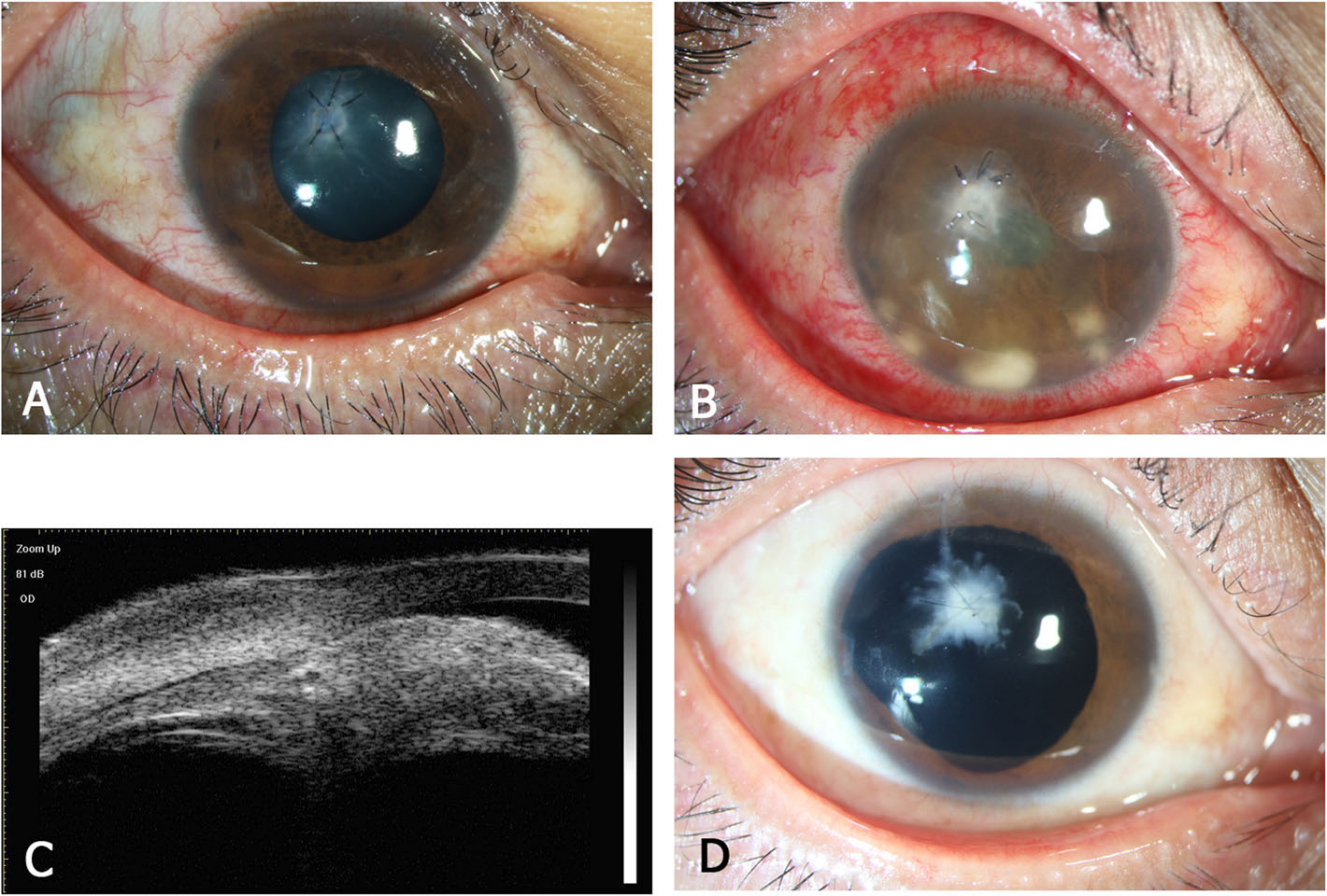
Examination images of patient no. 13. **a** Anterior segment photograph at 1 day after corneal repair; **b**. Anterior segment photograph at 35 days after corneal repair; **c**. Thirty-five days after corneal repair, ultrasound biomicroscopy showed a hyperechoic lesion at the temporal posterior iris. **d**. Eleven months after vitrectomy, an anterior segment photograph showed a central corneal scar and excision of the temporal iris

All 35 eyes underwent vitrectomy, which was combined with lensectomy at the first vitrectomy. Twenty-three eyes underwent one vitrectomy and 12 eyes underwent two vitrectomies. Silicone oil tamponade was applied in 14 eyes, and 10 eyes received silicone oil removal during follow-up. Four eyes underwent iridectomy because of the fungal lesion behind the iris. In one eye, iridectomy was performed promptly during the first vitrectomy (shown as a representative case in Fig. 1)**.** In the other three eyes, iridectomy was performed during the second vitrectomy.

An antifungal drug (amphotericin B or voriconazole) was injected into 19 eyes during the first vitrectomy. In the other 16 eyes, antibiotics (ceftazidime plus norvancomycin) were injected during the first vitrectomy. Of these 16 eyes, 6 eyes received supplementary injection of an antifungal drug, and 10 eyes underwent the second vitrectomy combined with supplementary injection of an antifungal drug. All affected eyes were treated with antifungal eye drops (natamycin or voriconazole eye drops). Oral antifungal drugs (itraconazole or voriconazole) were administered to 32 patients for at least one month, but not to the other three patients (all children). Table 2 lists the treatments and visual outcomes of patients with fungal endophthalmitis.

**Table 2:** Treatments and visual outcomes in patients with fungal endophthalmitis

Patient No.	VA at presentation	First PPV	Subsequent PPV or IVI	Final VA
1	HM	PPV, LE, IVI*	PPV, IE, SO, IVI (Vori)	HM
2	HM	PPV, LE, IVI*	PPV, SO, IVI (Vori)	HM
3	LP	PPV, LE, SO, IVI (Vori)	NA	HM
4	HM	PPV, LE, SO, IVI (AmB)	NA	LP
5	HM	PPV, LE, IVI*	IVI (Vori)	20/250
6	LP	PPV, LE, IVI (AmB)	NA	HM
7	HM	PPV, LE, IVI (Vori)	PPV, SO, IVI (Vori)	LP
8	LP	PPV, LE, IVI (Vori)	NA	LP
9	LP	PPV, LE, IVI (AmB)	NA	LP
10	LP	PPV, LE, IVI*	PPV, IVI (Vori)	LP
11	HM	PPV, LE, IVI (AmB)	NA	CF
12	HM	PPV, LE, IVI*	PPV, SO, IVI (Vori)	HM
13	LP	PPV, LE, IE, SO, IVI (Vori)	NA	20/200
14	LP	PPV, LE, IVI (AmB)	NA	HM
15	HM	PPV, LE, IVI (Vori)	NA	20/100
16	LP	PPV, LE, IVI*	IVI (AmB); enucleation	NLP
17	LP	PPV, LE, IVI*	IVI (AmB); PPV, IE, SO, IVI (AmB)	20/400
18	HM	PPV, LE, IVI (AmB)	NA	20/400
19	CF	PPV, LE, IVI*	IVI (AmB); PPV, IE, SO, IVI (AmB)	HM
20	LP	PPV, LE, SO, IVI (AmB)	NA	20/50
21	HM	PPV, LE, IVI*	PPV, IVI (AmB)	HM
22	LP	PPV, LE, IVI*	IVI (AmB)	CF
23	LP	PPV, LE, IVI*	PPV, SO, IVI (Vori)	HM
24	LP	PPV, LE, IVI (AmB)	NA	LP
25	LP	PPV, LE, IVI (AmB)	NA	CF
26	HM	PPV,LE,IVI (AmB)	NA	20/400
27	HM	PPV,LE,SO,IVI (AmB)	NA	20/250
28	HM	PPV,LE,IVI (Vori)	PPV,SO,IVI (Vori)	20/400
29	LP	PPV, LE, IVI*	PPV, SO, IVI (Vori)	LP
30	LP	PPV, LE, IVI (AmB)	NA	20/400
31	LP	PPV, LE, IVI*	IVI (AmB); enucleation	NLP
32	LP	PPV, LE, IVI*	IVI (Vori)	LP
33	HM	PPV, LE, IVI*	PPV, IVI (Vori)	20/160
34	HM	PPV, LE, IVI (AmB)	NA	20/40
35	LP	PPV, LE, IVI*	IVI (AmB)	LP

*VA* visual acuity, *IVI* intravitreal injection, *IVI antibiotics (ceftazidime + norvancomycin), *PPV* pars plana vitrectomy, *LE* lensectomy, *IE* iridectomy, *SO* silicone oil, *Vori* voriconazole, *AmB* amphotericin B, *NA* not applicable, *NLP* no light perception, *LP* light perception, *HM* hand motion, *CF* counting fingers

Fungal endophthalmitis was effectively controlled in 33 eyes (94.3%), whereas two eyes (5.7%) were ultimately enucleated. The final visual acuity was significantly better after treatment than the preoperative visual acuity (*P* = 0.0006) (Table 3). Fifteen eyes (42.9%) achieved a final vision of counting fingers or better, and twelve eyes (34.3%) achieved a final vision of 20/400 or better.

**Table 3:** Visual acuity before and after vitrectomy in eyes with fungal endophthalmitis

Time	NLP	LP	HM	CF	20/400 or better
Before surgery	0	19	15	1	0
After surgery	2	9	9	3	12

Values are number of eyes.

*NLP* no light perception, *LP* light perception, *HM* hand motion, *CF* counting fingers

We analyzed the factors that influenced the visual outcome. According to the preoperative vision, the affected eyes were divided into two groups (Table 4): group 1A (light perception) and group 1B (better than light perception). The final visual acuity in group 1B was significantly better than that in group 1A (*P* = 0.0289). According to whether an antifungal drug was injected during the first vitrectomy, the affected eyes were divided into two groups (Table 5): group 2A (antifungal drug injected during the first vitrectomy) and group 2B (antifungal drug not injected during the first vitrectomy). The final visual acuity in group 2A was better than that in group 2B, and the difference was near statistical significance (*P* = 0.0600). According to the latent period (time from trauma to endophthalmitis), the affected eyes were divided into two groups (**Supplemental Table** **1**): group 3A (< 1 month) and group 3B (> 1 month). There was no significant difference of visual outcome between group 3A and group 3B (*P* = 0.4428).

**Table 4:** Visual outcomes of eyes divided into two groups according to preoperative vision

Group	Preoperative vision	Final visual acuity					Total
		NLP	LP	HM	CF	20/400 or better	
Group 1A	LP	2	7	4	2	4	19
Group 1B	better than LP	0	2	5	1	8	16

Values are number of eyes.

*NLP* no light perception, LP light perception

**Table 5:** Visual outcomes of eyes divided into two groups according to whether an antifungal drug was injected during the first vitrectomy

Group	IVI antifungal drug in the first vitrectomy	Final visual acuity					Total
		NLP	LP	HM	CF	20/400 or better	
Group 2A	Yes	0	5	3	2	9	19
Group 2B	No	2	4	6	1	3	16

Values are number of eyes.

*IVI* intravitreal injection, *NLP* no light perception, *LP* light perception, *HM* hand motion, *CF* counting fingers

### Representative case

Here we describe a typical case of fungal endophthalmitis. The right eye of a 44-year-old man (patient no. 13) suffered cornea-penetrating trauma caused by iron scurf. The patient underwent emergency repair of the corneal perforation (Fig. 1a). Thirty-five days later, inflammatory infiltration was detected in the corneal wound. Corneal confocal microscopy revealed suspicious fungal hyphae. Multiple yellow-white mass lesions appeared in the anterior chamber, accompanied by severe intraocular inflammation (Fig. 1b). The affected eye was initially diagnosed with fungal endophthalmitis. Ultrasound biomicroscopy showed a posterior iris lesion on the temporal side (Fig. 1c). The affected eye was treated with vitrectomy combined with lensectomy, temporal iridectomy, and silicone oil tamponade. Voriconazole was injected into the vitreous cavity at the end of surgery. Culture of aqueous humor and vitreous specimens confirmed *Aspergillus* infection. After vitrectomy and systemic antifungal therapy (oral itraconazole), the patient’s endophthalmitis was effectively controlled (Fig. 1d). Silicone oil was removed 11 months after primary vitrectomy, and his final best-corrected visual acuity was 20/200.

## Discussion

Our study reported a large sample of consecutive cases of posttraumatic fungal endophthalmitis in Eastern China. Our results indicate that penetrating objects can cause fungal endophthalmitis, with plant material and iron objects being the predominant causes. As in previous studies [6–8], we observed a latent period of fungal endophthalmitis after a penetrating ocular trauma. The onset of fungal endophthalmitis after trauma was usually subacute or chronic. Up to 42.9% of cases of fungal endophthalmitis occurred > 1 month after ocular trauma. But, we did not observe the significant influence of latent period on the prognosis of visual acuity.

Chakrabarti et al. [6] retrospectively analyzed Indian patients with fungal endophthalmitis in a 14-year period and identified 23 cases of posttraumatic fungal endophthalmitis with positive culture results. Of these 23 patients, 14 (60.9%) were infected with *Aspergillus*, two (8.7%) with *Fusarium*, and three (13.0%) with yeast. Among the 35 cases in Eastern China in the present study, all were infected with filamentous fungi, including 26 (74.3%) with *Aspergillus* and one (2.9%) with *Fusarium*. No yeast infection was detected in our study. Generally, *Aspergillus* is the main cause of posttraumatic fungal endophthalmitis.

Fungal endophthalmitis usually presents with hypopyon, and the pus in the anterior chamber is always sticky. We also found that fungal endophthalmitis can present with mass lesions in the anterior chamber and that fungi can hide and grow in the space behind the iris. Vitrectomy can remove fungal pathogens and toxins from the vitreous and can be combined with other intraocular procedures, such as lensectomy, the removal of an intraocular foreign body, and silicone oil tamponade [11]. The indications for lensectomy include traumatic lens opacity and purulent exudation on the lens surface [5]. In cases of severe endophthalmitis, the inclusion of lensectomy during vitrectomy allows the inflammatory exudate in the vitreous cavity to be drained through the trabecular meshwork. Silicone oil can be used for fungal endophthalmitis with retinal necrosis. On the other hand, silicone oil has the potential role of inhibiting pathogenic microorganisms [12]. For serious intraocular infections which cannot be controlled by the first vitrectomy surgery, second vitrectomy combined with silicone oil tamponade is helpful to control the infection. If a fungal lesion is found behind the iris before or during vitrectomy, the iris can be excised to remove the infectious lesion completely [13]. In our case series, four eyes underwent iridectomy for a posterior iris fungal lesion and the intraocular fungal infection in the eyes was ultimately controlled.

Systemic delivery of therapeutic concentrations of antifungal drugs to the eye is difficult. However, intravitreal injection of antifungal drugs can improve the intraocular drug concentration. Amphotericin B and voriconazole inhibit and eliminate filamentous fungi, and are used intravitreally to treat fungal endophthalmitis [14–16]. Our retrospective analysis showed that an antifungal drug was injected during the first vitrectomy in 19 eyes and antibiotics were injected in the other 16 eyes. Because the incidence of bacterial infection is much higher than that of fungal infection after penetrating ocular trauma [17–19], clinicians prefer to treat posttraumatic endophthalmitis with antibiotics. In routine clinical practice, the treatment of fungal endophthalmitis may be delayed. In this study, we analyzed the effect of antifungal drugs given during the first vitrectomy on the prognosis of visual acuity. The difference of visual outcome between two groups was near statistical significance. We also noted that neither of two enucleated eyes was injected with an antifungal drug during the first vitrectomy. Therefore, the early diagnosis of fungal endophthalmitis and timely vitrectomy combined with an intravitreal injection of an antifungal drug can mitigate the devastating results of intraocular fungal infection.

Because of the difficulty of early diagnosis and the severe damage of the eye caused by fungal infection, the prognosis of fungal endophthalmitis is usually poor, especially after trauma. Wykoff et al. [9] reported that seven of 10 cases of posttraumatic fungal endophthalmitis finally underwent enucleation. In our study, 33 eyes (94.3%) with fungal endophthalmitis were effectively controlled, and these eyeballs were successfully preserved. However, two eyes were enucleated. The visual acuity of our patients improved significantly after treatment, and 42.9% of the patients gained useful vision (counting fingers or better). The main reason for the final poor visual acuity (worse than counting fingers) is the retinal damage caused by fungal infection. In this study, we analyzed the effect of the preoperative vision on the prognosis of visual acuity. We found that the preoperative visual acuity was a significant factor affecting the prognosis of visual acuity. When the preoperative visual acuity was better than light perception, surgical treatment can obtain better postoperative vision.

In conclusion, this study extends our understanding of the clinical features of fungal endophthalmitis. Vitrectomy combined with intravitreal antifungal therapy is an effective treatment for posttraumatic fungal endophthalmitis. Preoperative visual acuity is a significant factor affecting the prognosis of visual acuity.

## Supplementary Information

**Additional file 1 Supplemental table 1.** Visual outcomes of eyes divided into two groups according to the latent period (Time from trauma to endophthalmitis).

## Data Availability

The data used to support the findings of this study are available from the corresponding author upon request.

## Abbreviations

IVI: Intravitreal injection
PPV: Pars plana vitrectomy
VA: Visual acuity
NLP: No light perception
LP: Light perception
HM: Hand motion
CF: Counting fingers
