# Supplementary material for: Vitrectomy combined with intravitreal antifungal therapy for posttraumatic fungal endophthalmitis in eastern China
Source: BMC Ophthalmol. 2020 Nov 3;20:435. doi: 10.1186/s12886-020-01703-7 (PMC7607652; doi:10.1186/s12886-020-01703-7)
Supplement: Supplementary file 1 — Additional file 1 Supplemental table 1. Visual outcomes of eyes divided into two groups according to the latent period (Time from trauma to endophthalmitis). [file 12886_2020_1703_MOESM1_ESM.docx]

**Supplemental table 1.** Visual outcomes of eyes divided into two groups according to the latent period (Time from trauma to endophthalmitis).

| Group | Time from trauma to endophthalmitis | Final visual acuity | | | | | Total |
| --- | --- | --- | --- | --- | --- | --- | --- |
|  |  | NLP | LP | HM | CF | 20/400 or better |  |
| Group 3A | < 1 month | 0 | 5 | 6 | 2 | 7 | 20 |
| Group 3B | > 1 month | 2 | 4 | 3 | 1 | 5 | 15 |

Values are number of eyes

NLP: no light perception; LP: light perception; HM: hand motion; CF: counting fingers
